# Impact of RhoA overexpression on clinical outcomes in cervical squamous cell carcinoma treated with concurrent chemoradiotherapy

**DOI:** 10.1093/jrr/rrz093

**Published:** 2020-01-24

**Authors:** Keiichi Tanaka, Yoshitaka Matsumoto, Hitoshi Ishikawa, Nobuyoshi Fukumitsu, Haruko Numajiri, Keiko Murofushi, Yoshiko Oshiro, Toshiyuki Okumura, Toyomi Satoh, Hideyuki Sakurai

**Affiliations:** 1 Proton Medical Research Center, University of Tsukuba, 2-1-1 Amakubo, Tsukuba, Ibaraki 305-8576, Japan; 2 Obstetrics & Gynecology of University of Tsukuba, 1-1-1 Tennodai, Tsukuba, Ibaraki 305-8575, Japan; 3 Department of Radiation Oncology, Kobe Proton Center, 1-6-8, Minatoshima-minamimachi, Chuou-ku, Kobe, 650-0047, Japan

**Keywords:** cervical cancer, prognosis, concurrent chemoradiotherapy, RhoA, cell adhesion

## Abstract

The Rho-associated coiled-coil-containing protein kinase (ROCK) pathway is known to influence metastasis in several cancers; however, the impact of the pathway on clinical outcomes in patients undergoing radiotherapy remains unknown. In the present study, the expression of RhoA, RhoC, ROCK-1, ROCK-2 and p53 was immunohistochemically evaluated using biopsy specimens obtained from 49 patients with stage II–III cervical squamous cell carcinoma treated with concurrent chemoradiotherapy (CCRT). The relationship between the expression of these proteins and patient outcomes was investigated. RhoA overexpression was associated with significantly impaired disease-free survival and distant metastasis-free survival (*P* = 0.045 and *P* = 0.041, respectively) in stage III cancer patients. No differences in survival were observed based on the expression of the other proteins among stage III cancer patients. In stage II cancer patients, no differences in survival were noted based on the expression of any of the proteins. The expression of RhoA was able to successfully differentiate cervical cancer patients with distant metastasis after CCRT. This information may help stratify patients according to the risk of metastasis, thereby leading to the potential to provide individualized treatment.

## INTRODUCTION

Concurrent chemoradiotherapy (CCRT) for cervical cancer results in better tumor control than radiotherapy (RT) alone. A randomized trial showed that locoregional control was obtained in 81% of patients after CCRT, whereas only 65% of patients receiving RT alone achieved locoregional control, and the overall survival (OS) rates in the CCRT and RT alone groups were 73 and 58%, respectively [1]. In addition, advances in RT techniques, especially in terms of image-guided brachytherapy for cervical cancer, have contributed to favorable local tumor control and survival rates [2]. However, survival is often decreased due to recurrence and distant metastasis, especially in advanced cervical cancer. A meta-analysis indicated the benefits of adjuvant chemotherapy after CCRT in improving survival, mainly through the suppression of the progression of invisible distant metastases [3]. It is, therefore, important for physicians to determine which patients will benefit from adjuvant chemotherapy after CCRT. However, useful biomarkers for the classification of patients who require adjuvant chemotherapy have not yet been identified.

Rho GTPases belong to the RAS superfamily. They control the dynamics of the actin cytoskeleton and play a role in cell metastatic potential, including migration and invasion ability [4, 5]. Several previous reports have supported a relationship between the overexpression of Rho GTPases and tumor invasion and metastasis. For example, one study showed a correlation between RhoA GTPase overexpression and cell motility in breast cancer [6], and another demonstrated that the coexpression rate of delta-catenin, which regulates the cytoskeleton and RhoA expression, was stronger in stage III–IV lung cancer than in stage I–II lung cancer [7].

Rho-associated coiled-coil-containing protein kinase (ROCK) is an effector of the Rho GTPases [8]. ROCK activation downstream of Rho GTPases is implicated in multiple cellular processes, such as motility, morphogenesis, polarity, cell division and cell adhesion [9]. In humans, two isoforms of ROCK, ROCK1 and ROCK2, have been identified. ROCK overexpression has been implicated in the progression of several cancers, such as bladder, laryngeal and breast cancers [10–12]. The therapeutic potential of decreased expression or inhibition of ROCK activity has been described in some types of cancers, such as prostate cancer [13]. The Rho–ROCK pathway is one of the key pathways pertaining to metastasis, and the expression levels of Rho and/or ROCK are positively correlated with tumor grade and distant metastasis [10–13].

Although Rho GTPases and ROCK are closely related to tumor progression in various cancers, the impact of Rho GTPases on clinical outcomes in cervical cancer remains unclear. The purpose of this study was to analyse the relationship between the expression of Rho GTPases and ROCK proteins and clinical results in cervical cancers after CCRT.

## MATERIALS AND METHODS

### Patients

A total of 476 patients with squamous cell carcinoma (SCC) of the uterine cervix were treated with definitive CCRT using cisplatin (CDDP) from March 2008 to June 2014 in our institution. Because most patients with stage IB undergo surgery without CCRT and stage IV patients have a poor prognosis, these patients were excluded. Of the remaining patients, 49 had available tumor biopsy specimens before CCRT and were evaluated in the present study. All 49 included patients had stage II or III SCC according to the International Federation of Gynecology and Obstetrics classification 2008 (FIGO 2008) [14]. Tumor staging was determined by the radiologist’s report and the judgment of a clinical conference in our institute based on the Response Evaluation Criteria in Solid Tumors Group guideline v1.1 [15]. The patients’ characteristics are summarized in Table 1. The median age of the patients was 55 years (range, 27–75 years) at the start of treatment. A total of 41 patients had regional lymph node metastasis and 5 had para-aortic lymph node (PALN) metastasis. The median size of the lymph nodes diagnosed as positive was 18.8 mm (range, 8–74 mm). All patients were periodically followed up at monthly intervals by a gynecological examination for up to 5 years, and computed tomography and/or magnetic resonance imaging were performed in patients with symptoms or suspicions of residual tumor presence. The median follow-up time was 40.1 months (range, 2.3–80.1 months) as of January 2016 at the time of the analysis. This study has been approved by the ethics review board of the University of Tsukuba Hospital (H26–054), and all patients provided consent.

**Table 1:** Characteristics of patients and treatment

**Characteristics**	**Number of patients (%)**
Age (years)	
<59	30 (61)
60–69	17 (35)
≥70	2 (4)
Performance status	
0	37 (76)
1	11 (22)
2	1 (2)
FIGO stage	
IIA	2 (4)
IIB	7 (14)
IIIA	2 (4)
IIIB	38 (78)
LN swelling	
Positive	41 (84)
Negative	8 (16)
Chemotherapy	
4 courses	5 (10)
5 courses	44 (90)
Radiotherapy (extra)	
WP 30 Gy + CS 20 Gy	43 (88)
WP 40 Gy + CS 10 Gy	4 (8)
WP 45 Gy + CS 5.4 Gy	2 (4)
Radiotherapy (RALS)	
12 Gy/2 fr	1 (2)
24 Gy/4 fr	42 (86)
30 Gy/5 fr	6 (12)

FIGO = International Federation of Gynecology, LN = lymph node, WP = whole pelvis, CS

= center shield, RALS = remote after loading system, fr = fraction.

### Treatment

The treatment policy in our institute includes the provision of external beam RT (EBRT) to the entire pelvis combined with four to five sessions of high-dose-rate remote after-loading intracavitary brachytherapy (HDR-IB). Radiation beams were administered to the entire pelvis with the patient in the prone position using either a four-field box or an anterior and posterior opposing two-field irradiation technique with 10-MV X-rays from a linear accelerator (Varian iX; Varian, Palo Alto, CA, USA). A total dose of 50 Gy in 25 fractions with conventional fractionation was administered to the pelvis and a central shielding (3 cm in width) was inserted at 30 Gy (4 patients were treated with 40 Gy in 20 fractions and central shielded with 10 Gy and 2 patients were treated with 45 Gy and central shielded with 5.4 Gy by the decision of the attending doctor). Extended EBRT fields were applied for patients who required irradiation to the PALNs after the insertion of the midline shield in whole pelvis RT to avoid overdoses to the gastrointestinal tract. The modified Manchester system was used for HDR-IB, with an ^192^Ir source (Microselectron; Elekta, Stockholm, Sweden). Each brachytherapy session delivered a dose of 6.0 Gy at point A. There was at least a 7-day interval between each brachytherapy session. Weekly CDDP (40 mg/m^2^/course) was concurrently combined with RT. Carboplatin was administered instead of CDDP in one patient because of cardiac failure. The overall treatment time was <6 weeks in most patients.

### Specimens and immunohistochemistry staining

Immunohistochemistry was performed using a polymer-based detection system to examine the expression of p53 for tumor characteristics and those of RhoA, RhoC, ROCK-1 and ROCK-2 for metastatic ability.

Specimens were obtained from all 49 patients before CCRT, and they were fixed with 10% neutral-buffered formalin and embedded in paraffin blocks. Next, 2-μm thick sections were cut from the blocks for immunohistochemical staining and dried at 65°C for 1 h and deparaffinized with 100% xylenes for 5 min. They were then rehydrated and incubated with fresh ethanol at room temperature. After rehydration through a graded ethanol series, the tissue sections were autoclaved in zinc citrate buffer (pH = 6.0, Muto Pure Chemicals, Tokyo, Japan) at 115°C for 10 min and then quenched at 105°C for 10 min with Dako Target Retrieval Solution pH 9 (Dako, Glostrup, Denmark) at a 1:10 dilution. Peroxidase-blocking solution (S2023, Dako) was applied to block endogenous peroxidase activity for 5 min and then washed with buffer (S3006, Dako). The sections were incubated with each primary monoclonal antibody at the following dilutions: 1:100 for human p53 (DO-7) (mouse) (M700101, Dako), 1:100 for RhoA (rabbit) (10749–1-AP, Proteintech, USA), 1:50 for RhoC (rabbit) (ABG AP14105B, Abgent, USA), 1:100 for ROCK-1 (rabbit) (HPA007567, Atlas Antibodies, Sweden) and 1:200 for ROCK-2 (rabbit) (HPA007567, Atlas Antibodies).

Thereafter, the sections were washed with phosphate-buffered saline and incubated with secondary antibodies (EnVIsion+ K4061, Dako) for 30 min. Immunohistochemistry was performed using the Liquid DAB+ Substrate Chromogen System (K3468, Dako), which entails the use of 1 mL of DAB+ substrate buffer and 1 drop of DAB+ chromogen for 5 min, followed by washing with buffer. The sections were counterstained with hematoxylin (Tissue-tek Hematoxylin 3G, Sakura Finetek Japan Co., Japan) for 5–10 s.

**Fig. 1.**
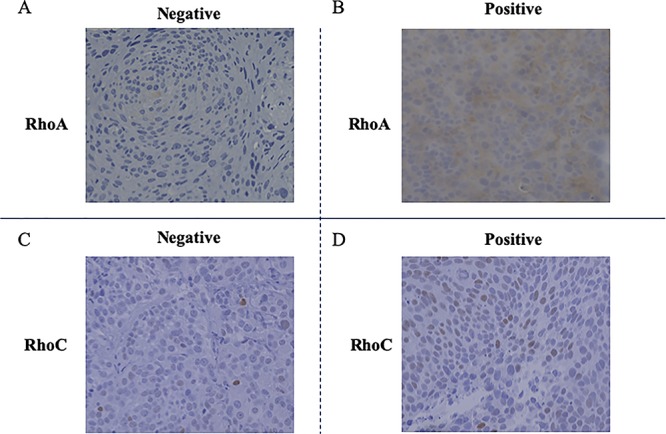
Representative specimens of (**A**) negative and (**B**) positive RhoA expression. Representative specimens of (**C**) negative and (**D**) positive RhoC expression. For samples in which the cytoplasm was stained, (A) negative expression (score 1) was defined as faint intensity (+) and <25% positively stained cells, and positive expression (score 8) was defined as moderate intensity (++) and >75% positively stained cells. For samples in which the nucleus was stained, (C) negative expression was defined as <25% positively stained cells, and (D) positive expression was defined as >25% positively stained cells.

Each stained section was observed under a BZ-X710 microscope (KEYENCE, Japan) and five independent photographs were taken for each sample. The intensity of cytoplasmic staining was classified as strong (+++), moderate (++), faint (+) or negative (−), with reference to a positive internal control (myoepithelial or vascular smooth muscle cells). Strong intensity (+++) was given a score of 3, moderate (++) a score of 2, faint (+) a score of 1 and negative (−) a score of 0. The proportion of positive cells was classified as 1–4 using 25% intervals, as follows: samples with 0–25% positive cells were given a score of 1, those with 26–50% positive cells a score of 2, those with 51–75% positive cells a score of 3 and those with 76–100% positive cells a score of 4. The immunohistochemistry score was calculated by multiplying the intensity score by the positivity score. Samples were considered positive for RhoC with a score of 6 or higher and for RhoA, ROCK-1 and ROCK-2 with a score of 4 or higher. For example, if a specimen immunohistochemically stained for RhoA had moderate intensity (intensity score = 2) and the proportion of positive cells was 60% (positivity score = 3), the total score of this specimen would be 6, and it would be defined as positive. We judged p53 expression as being negative if the proportion of positive cells was <10% and positive if the value was ≥10% [16]. These rules of immunohistochemical classification are based on those from previous studies [11, 16, 17]. The evaluation of immunostaining was performed by three independent observers (K.T., Y.M. and H.I.). In the case of different scores, the judgment of the most experienced investigator (H.I.) was adopted as a final consensus. Representative findings of the immunohistochemical analysis are shown in Fig. 1.

### Statistical analysis

All statistical analyses were performed with the SPSS statistical software package (Ver.22.0; SPSS Inc., Chicago, IL, USA). Patient samples were classified as positive and negative based on RhoA, RhoC, ROCK-1, ROCK-2 and p53 expression. OS, disease-free survival (DFS), local control (LC) and distant metastasis-free survival (DMFS) rates were calculated using the Kaplan-Meier method. Differences in these rates between the positive and negative groups were evaluated by a log-rank test. Differences were considered significant at *P* < 0.05.

## RESULTS 

### Recurrence and survival

The 3-year OS, DFS, LC and DMFS rates with 95% confidence interval (CI) the of entire cohort were 88.4, 65.9, 88.4 and 73.2%, respectively. In stage II patients, the 3-year OS, DFS, LC and DMFS rates with 95% CI were 100.0, 63.5, 100.0 and 76.2%, respectively. In stage III patients, the 3-year OS, DFS, LC and DMFS rates with 95% CI were 90.5, 65.6, 88.1 and 74.0%, respectively.

At the last follow-up, 33 patients were alive without disease, 9 patients were alive with recurrence, 5 patients died of tumor recurrence and 2 patients died of another disease (1 patient had tumor recurrence and the other patient had no tumor recurrence) (Table 2).

**Table 2:** Sites of recurrence

	Locoregional (%)	Distant (%)	All (%)
49 Patients (stage II and III)			
Recurrence: Yes	6 (12)	12 (24)	15 (31)
No	43 (88)	37 (76)	34 (69)
9 Patients (stage II)			
Recurrence: Yes	1 (11)	2 (22)	3 (33)
No	8 (89)	6 (67)	6 (67)
40 Patients (stage III)			
Recurrence: Yes	5 (13)	9 (23)	11 (28)
No	35 (88)	31 (78)	29 (73)

**Table 3:** Prognostic factors of stage III cervical cancer

Factors			OS		DFS		LCR		DMFR	
			percentage	*P* value	percentage	*P* value	percentage	*P* value	percentage	*P* value
49 Patients (stages II and III)										
RhoA										
	Positive	*n* = 28	88	0.755	58	0.102	80	0.132	66	0.121
	Negative	*n* = 21	87		80		100		84	
RhoC										
	Positive	*n* = 34	91	0.629	63	0.631	89	0.474	72	0.673
	Negative	*n* = 15	90		72		87		79	
ROCK-1										
	Positive	*n* = 36	94	0.122	67	0.541	90	0.895	76	0.527
	Negative	*n* = 13	82		61		84		69	
ROCK-2										
	Positive	*n* = 24	92	0.882	69	0.692	87	0.988	83	0.294
	Negative	*n* = 25	90		62		88		66	
p53										
	Positive	*n* = 9	83	0.822	62	0.899	89	0.851	62	0.761
	Negative	*n* = 40	92		66		88		76	
9 Patients (stage II)										
RhoA										
	Positive	*n* = 3	100	0.480	100	0.237	100	0.480	100	0.343
	Negative	*n* = 6	100		50		100		67	
RhoC										
	Positive	*n* = 7	100	0.655	51	0.277	100	0.655	69	0.408
	Negative	*n* = 2	100		100		100		100	
ROCK-1										
	Positive	*n* = 7	100	0.025	69	0.650	100	0.025	69	0.408
	Negative	*n* = 2	50		50		100		100	
ROCK-2										
	Positive	*n* = 3	100	0.655	67	0.953	100	0.655	67	0.560
	Negative	*n* = 6	100		60		100		80	
p53										
	Positive	*n* = 1	100	NS	100	0.483	100	NS	100	0.589
	Negative	*n* = 8	100		58		100		73	
40 Patients (stage III)										
RhoA										
	Positive	*n* = 25	84	0.508	52	0.045	81	0.097	62	0.041
	Negative	*n* = 15	93		86		100		92	
RhoC										
	Positive	*n* = 27	88	0.810	61	0.452	91	0.436	68	0.390
	Negative	*n* = 13	88		77		85		85	
ROCK-1										
	Positive	*n* = 29	93	0.350	67	0.712	87	0.876	77	0.329
	Negative	*n* = 11	78		64		91		64	
ROCK-2										
	Positive	*n* = 21	91	0.985	70	0.653	87	0.947	85	0.165
	Negative	*n* = 19	87		62		90		62	
p53										
	Positive	*n* = 8	80	0.715	56	0.705	88	0.731	56	0.393
	Negative	*n* = 32	90		68		89		77	

**Fig. 2.**
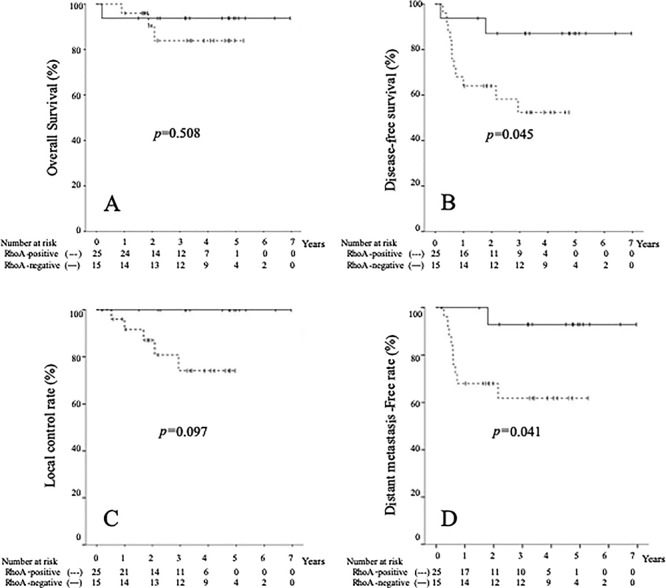
Kaplan-Meier curves of patients with stage III cervical squamous cell carcinoma according to RhoA expression. Overall survival (OS) (**A**), disease-free survival (DFS) (**B**), local control rate (LCR) (**C**), and distant metastasis-free survival (DMFS) (**D**). The solid line represents RhoA-positive patients and the dotted line represents RhoA-negative patients.

**Table 4:** Correlation between RhoA expression and patient characteristics

Factors		RhoA expression			
		*n* (%)	Positive	Negative	*P* value
FIGO stage					
	II	9	3	6	0.221
	III	40	25	15	
Tumor size					
	≤4 cm	9	6	^3^	0.688
	>4 cm	40	21	19	
LN swelling					
	Positive	41	23	18	1.000
	Negative	8	4	4	

LN = lymph node.

### Relationship between protein expression and treatment outcomes

Table 3 summarizes the results pertaining to protein expression in the present study. RhoA overexpression was correlated with significantly worse DFS and DMFS rates (*P* = 0.045 and *P* = 0.041, respectively) in stage III cancer patients (Table 3 and Figure 2). No other significant findings were observed in stage III patients. There was no significant correlation between survival and expression of any of the proteins in stage II patients.

Of the RhoA-positive stage III cancer patients, 11 (44%) of 25 patients experienced recurrence. The recurrence pattern was locoregional in 5 patients and distant metastasis in 9; a combination of the two was observed in 3 patients. The metastasis site was the lung in 4 patients, the bone in 1 patient, the pelvic lymph node in 2 patients, the PALN in 3 patients, other lymph nodes in 3 patients, the brain in 1 patient, the vagina in 1 patient, the uterus in 1 patient and the liver in 1 patient. In contrast, in the RhoA-negative stage III cancer patients, 1 (6.7%) of 15 patients developed PALN recurrence. The recurrence pattern was distant metastasis in 1 patient; the metastasis site was the lung. The 3-year DFS and DMFS rates were 90.5 and 65.6%, respectively.

Statistically, the expression of RhoA did not correlate with other patient features, such as local progression (FIGO stage), tumor size (>4 cm or ≤4 cm), lymph node category or expression of other Rho-related proteins (Table 4 and 5). In addition, because it has been reported that RhoC can be used as a biomarker for judging the metastatic potential of tumors [18], we examined the correlation between double positivity of RhoA and RhoC and clinical outcomes. There was no significance in the OS, DFS, LC or DMFS rates based on RhoA and RhoC double positivity in the entire patient cohort (*P* = 0.352, 0.161, 0.331 and 0.222, respectively). Even when focusing on only the 40 patients with stage II disease, there was no correlation (*P* = 0.406, 0.061, 0.361 and 0.089, respectively).

**Table 5:** Correlation between RhoA expression and the expressions of other proteins

49 Patients (stages II and III)				
		RhoA (%)		
		Positive (*n* = 28)	Negative (*n* = 21)	*P-*value
RhoC				
	Positive	21 (43)	14 (29)	0.102
	Negative	7 (14)	7 (14)	
ROCK-1				
	Positive	20 (41)	15 (31)	1.000
	Negative	8 (16)	6 (12)	
ROCK-2				
	Positive	14 (29)	10 (20)	1.000
	Negative	14 (29)	11 (22)	
p53				
	Positive	5 (10)	4 (8)	1.000
	Negative	23 (47)	17 (35)	
9 Patients (stage II)				
		RhoA (%)		
		Positive (*n* = 3)	Negative (*n* = 6)	*P-*value
RhoC				
	Positive	2 (22)	5 (56)	1.000
	Negative	1 (11)	1 (11)	
ROCK-1				
	Positive	3 (33)	4 (44)	0.777
	Negative	0 (0)	2 (22)	
ROCK-2				
	Positive	1 (11)	2 (22)	1.000
	Negative	2 (22)	4 (44)	
p53				
	Positive	0 (0)	1 (11)	1.000
	Negative	3 (33)	5 (55)	
40 Patients (stage III)				
		RhoA (%)		
		Positive (*n* = 25)	Negative (*n* = 15)	*P-*value
RhoC				
	Positive	19 (48)	9 (23)	0.476
	Negative	6 (15)	6 (15)	
ROCK-1				
	Positive	17 (43)	11 (28)	1.000
	Negative	8 (20)	4 (10)	
ROCK-2				
	Positive	13 (33)	8 (20)	1.000
	Negative	12 (30)	7 (18)	
p53				
	Positive	5 (13)	3 (8)	1.000
	Negative	20 (50)	12 (30)	

## DISCUSSION

In the present study, we immunohistochemically evaluated the expression of RhoA, RhoC, ROCK-1, ROCK-2 and p53 in biopsy specimens obtained from 49 patients with stage II–III cervical squamous cell carcinoma treated with CCRT, and the relationships between the expression of these proteins and outcomes were investigated. We found that the expression of RhoA plays a key role in the prediction of distant metastasis after CCRT in such patients.

According to previous reports, RhoA expression is correlated only with N category (the absence or presence and extent of regional lymph node metastasis) and lymphatic invasion in gastric cancer patients [19, 20]. Further, overexpression of RhoA is associated with capsule invasion [21], portal vein invasion, dissemination [22], venous invasion and microscopic satellite legions [23] in hepatocellular carcinomas. Faried *et al.* reported that RhoA overexpression correlated with N and M category (the absence or presence of distant metastasis), lymphatic invasion and vascular invasion in esophageal SCC [24]. Although the tumor type was different from those examined in past studies, in our study, RhoA expression was higher in patients with more advanced stage disease and in those with metastasis. Our study is the first to demonstrate that the expression of RhoA is closely related to distant metastasis after CCRT in advanced-stage cervical cancer. An *in vitro* experiment showed that RhoA is involved in tumor migration via the VEC (Vascular endothelial growth factor C, VEGF-C)–RhoA–ROCK2 signaling pathway and correlates with cervical cancer metastasis [25]. In addition, all previous reports showed that there was no correlation between RhoA expression and age, gender, tumor size or tumor number, and those results are consistent with our result. In our study, there was no correlation between RhoA expression and local progression (FIGO stage), tumor size (>4 cm or ≤4 cm) or N category (*P =* 0.110, 0.440 and 0.751, respectively; chi-square test).

Only DMFS showed a correlation with RhoA expression in our study. However, previous reports indicated that RhoA was correlated with OS [19–22, 24], DFS [19], T category (the extent of the primary tumor) [20, 24], TNM stage (international cancer classification system defined by UICC (Union for International Cancer Control)) [20, 22–24, 26] and tumor differentiation [21, 22, 27, 28]. It has been suggested that RhoA expression may correlate not only with distant metastases, but also with local tumor progression and other factors such as OS and DFS. To clarify whether RhoA and related signaling pathways are independent predictors of metastasis, it is necessary to conduct analysis with a larger number of cases and to add basic experiments using cells and animal models.

Of all the evaluated proteins, only RhoA was associated with prognosis; no such correlation was observed for the other proteins. This could be because RhoA and RhoC have different downstream effectors. RhoA regulates ROCK1/2 to regulate the actin cytoskeleton and cell migration. In contrast, RhoC affects FMNL3, which regulates lamellipodium extension for cell migration and invasion of the cell. It is possible that these different downstream effectors underlie the disparity we observed in the relationships of survival to RhoA and the other proteins we assessed [29].

RhoA expression was correlated to DFS and DMFS, but not OS or LC. This indicates that although RhoA regulates the migration and invasion of cancer cells, which lead to distant metastasis, it does not significantly affect recurrence. The treatment of distant metastasis is very important not only in terms of prognosis but also for quality of life. In most cases of distant metastasis, treatment options are limited to intensive and systematic chemotherapy or palliation. RhoA has the potential to predict the occurrence of distant metastasis. If a high risk of distant metastasis can be predicted before CCRT, patients can be stratified accordingly, leading to the possibility to provide individualized treatment, such as adjuvant chemotherapy. In cervical cancer, the development of a new treatment protocol based on the presence of RhoA expression is highly expected. Mabuchi *et al*. reported that adjuvant paclitaxel plus carboplatin (TC)-based chemotherapy after concurrent CCRT improves OS in stage IIIB–IVA cervical cancer patients [30]. However, Tangjitgamol *et al*. reported that adjuvant TC-based chemotherapy improves systemic recurrence but does not improve OS or PFS for cervical cancer patients, including stage IIB–IVA patients [31]. In addition, a phase III study that aimed to determine whether adjuvant chemotherapy improves OS for advanced cervical cancer patients is underway [32]. Our study may also help to clarify suitable cohorts for adjuvant chemotherapy after CCRT.

The present study has some limitations. First, only nine of the patients in our study had stage II disease; this is many fewer patients than had stage III disease. We first conducted detailed comparisons of the expression of the Rho GTPase family proteins without considering the cancer stage. However, it may not be accurate to state that we compared Rho GTPase family activity levels between the clinical stages because of the small number of stage II patients who received CCRT during the defined research period. However, the statistical analysis showed that the difference between these biomarkers was far from significant. Moreover, a recent study showed that early-stage lung cancer is associated with weaker Rho protein expression [7]. Taken together, the Rho–ROCK pathway has weak potential to affect distant metastasis in early-stage cervical cancer. Second, it was difficult to count the number of cells, as Rho and ROCK are distributed in the cytoplasm. We calculated positivity based on the methods used in previous reports [11, 16, 17]. Subjective evaluation was included to determine a score that could reduce the objectivity of the evaluation. Further, the evaluation was performed by three physicians, including an experienced researcher, and we managed to maintain objectivity. Third, we examined the expression of the Rho GTPase family and p53 in this study. We found that RhoA expression is correlated with cervical cancer metastasis. In malignant tumors, hypoxia is predictive of treatment resistance, and it is known that hypoxia inducible factor-1 expression is correlated to DMFS in cervical cancer [33]. Further studies including other proteins should be conducted to elucidate the cellular and molecular mechanisms associated with distant metastasis in cervical cancer. Fourth, this study was a single-institutional retrospective analysis. In order to draw a more universal conclusion, treatment and analysis using a multicenter joint protocol is essential, and we would like to make use of it in future research planning. Fifth, *P*-values for ROCK-1 in relation to OS and LCR in stage II patients are 0.025 (Table 3), and it seems this is statistically significance. However, there are only nine patients with stage II cervical cancer in total in this research. This number of patients is not enough for reliable statistical analysis. We think that a much larger number of cases is required in further research.

In conclusion, in this study, we found that the expression of RhoA plays a key role in the prediction of distant metastasis after CCRT in cervical cancer patients. This information may help in the stratification of patients according to the risk of metastasis, thereby leading to the provision of individualized treatment.
